# Gender and age differences in the recurrence of sickness absence due to common mental disorders: a longitudinal study

**DOI:** 10.1186/1471-2458-10-426

**Published:** 2010-07-20

**Authors:** Petra C Koopmans, Corné AM Roelen, Ute Bültmann, Rob Hoedeman, Jac JL van der Klink, Johan W Groothoff

**Affiliations:** 1 ArboNed Occupational Health Services, Statistics, Groningen, The Netherlands; 2 Department of Health Sciences, University Medical Center Groningen, University of Groningen, The Netherlands; 3 ArboNed Occupational Health Services, Corporate Accounts, Zwolle, The Netherlands; 4 ArboNed Occupational Health Services, Corporate Accounts, Utrecht, The Netherlands

## Abstract

**Background:**

Common mental disorders (CMDs) are an important cause of sickness absence and long-term work disability. Although CMDs are known to have high recurrence rates, little is known about the recurrence of sickness absence due to CMDs. The aim of this study was to investigate the recurrence of sickness absence due to CMDs, including distress, adjustment disorders, depressive disorders and anxiety disorders, according to age, in male and female employees in the Netherlands.

**Methods:**

Data on sickness absence episodes due to CMDs were obtained for 137,172 employees working in the Dutch Post and Telecommunication companies between 2001 and 2007. The incidence density (ID) and recurrence density (RD) of sickness absence due to CMDs was calculated per 1000 person-years in men and women in the age-groups of < 35 years, 35-44 years, 45-54 years, and ≥ 55 years.

**Results:**

The ID of one episode of CMDs sickness absence was 25.0 per 1000 person-years, and the RD was 76.7 per 1000 person-years. Sickness absence due to psychiatric disorders (anxiety and depression) does not have a higher recurrence density of sickness absence due to any CMDs as compared to stress-related disorders (distress and adjustment disorders): 81.6 versus 76.0 per 1000 person-years. The ID of sickness absence due to CMDs was higher in women than in men, but the RD was similar. Recurrences were more frequent in women < 35 years and in women between 35 and 44 years of age. We observed no differences between age groups in men. Recurrences among employees with recurrent episodes occurred within 3 years in 90% of cases and the median time-to-onset of recurrence was 11 (10-13) months in men and 10 (9-12) months in women.

**Conclusions:**

Employees who have been absent from work due to CMDs are at increased risk of recurrent sickness absence due to CMDs and should be monitored after they return to work. The RD was similar in men and in women. In women < 45 years the RD was higher than in women ≥ 45 years. In men no age differences were observed.

## Background

Mental disorders are common in the general population, particularly among women between 25 and 45 years of age [1-4], and there is a high risk of recurrence. For depressive disorders, recurrences have been reported in 50-85% of patients, and very few demographic or clinical characteristics have been found to predict a recurrence after recovery from a major depressive disorder [5,6]. Recently, it has been reported that the more recurrences of depressive episodes, the higher the likelihood of a subsequent episode [7]. Depressive episodes remit but may leave psychological scars, such as negative cognitive patterns which were not present prior to the episode [8]. Such cognitive scars can also be caused by anxiety disorders, and may contribute to the recurrence of mental disorders [9-11]. Patients with two or more lifetime episodes of mental disorders, are at risk for long-term pharmacotherapy, especially if they have comorbid disorders, report psychosocial stressors, poor symptom control, or severe symptoms [12]. Despite pharmacotherapy, a chronic clinical course with low rates of recovery and a high probability of recurrence is found in the majority of patients suffering from anxiety disorders [13,14].

Common mental disorders (CMDs) include criteria-based psychiatric disorders, mostly mild to moderate depressive disorders and anxiety disorders, as well as 'subtreshhold'syndromes such as distress and adjustment disorders. CMDs are the second most frequent cause of sickness absence after musculoskeletal disorders [15-19]. In Scandinavian countries, the incidence of sickness absence due to CMDs is increasing when compared to sickness absence due to other diagnoses [17,18]. In the Netherlands a quadratic trend was observed: the 12-month incidence of sickness absence due to CMDs was 2.2% in 2001, increased to 2.7% in 2004 and decreased thereafter to 2.0% in 2007 [19]. Sickness absence due to CMDs has been associated with long-term work disability resulting in high social and economic costs [20-25]. Research on sickness absence among employees with psychiatric diagnoses has focused on the duration of the episodes of sickness absence and the risk of a disability pension. Vaez et al. found that 65% of those who returned to work after being absent due to mental disorders had high levels of all cause sickness absence in the following three years [21]. We assume that there is a high risk of recurrent sickness absence due to CMDs in employees who have returned to work after a previous episode of absence due to CMDs.

Although mental disorders are known to have high recurrence rates [5,6,14], the recurrence of sickness absence due to CMDs by gender and age has not been investigated yet. With regard to gender and sickness absence due to mental disorders contradictory findings are reported [26]. Women show higher sickness absence and are more frequently sick-listed due to mental disorders than men [27]. Although mental disorders are more common among women, sickness absence seems to be longer among male employees with mental disorders than among female employees with mental disorders [28,29]. However, Koopmans et al. [30] found a longer duration of absence due to depression in women than in men. Men and women had an equal duration until return to work (RTW), but women did report a longer time until lasting return to work than men after a long-term absence due to mental disorders [31]. Lasting return to work was defined as return to work without recurrence during the period in which the study was conducted. In the Netherlands the incidence of CMDs is higher in people aged 18-45 years than in those older than 45 years [2,32-34]. In the present study, the recurrence of sickness absence due to CMD will be examined, thereby stratifying by gender and age. In addition, the time-to-onset of recurrent episodes of sickness absence due to CMDs will be investigated. We hypothesize that the risk of recurrent sickness absence due to CMDs increases after a previous episode of sickness absence due to CMDs. We further hypothesize that women and employees < 45 years have a higher risk of recurrence than men and employees > 45 years.

## Methods

### Study population

This dynamic population study included employees who worked in the Dutch Post and Telecommunication companies in the period from 2001 to 2007. Employees were included on January 1st 2001; employees who started working later were included on the date they entered employment, and employees who resigned or retired during the study period were censored on the day they left employment. Employees who reached the one-year sickness absence date were also censored on that date. The total population consisted of 137,172 employees (62% men and 38% women), with a total of 363,461 person-years at risk. Approximately 70% of the employees worked as post sorters, postmen and post officers in the Post companies. The remainder of 30% worked in the Telecommunication companies, and were charged with installing and maintaining telephone, telefax and internet systems or worked in the call center or client services. Many employees, especially those in the Post companies, worked on a temporary basis. Both companies had a high turnover. Of the 67,316 employees who were included in the study on January 1^st^, 2001, 51,280 resigned or were discharged and right censored on the date they left employment, or their company left our occupational health department. A total of 69,856 employees started working for the Post and Telecommunication companies after January 1^st^, 2001 and were included on the date they entered employment. Of those 48,231 left their job again or their company left our occupational health department before the end of the study period and these employees were right censored on the leaving date.

### Ethical approval

The Medical Ethics Committee of the University Medical Center in Groningen informed us that ethical approval was not required because the data were analysed in retrospect at group level.

### Sickness absence in the Netherlands

Since 2004 most employers pay 100% of the income of an employee in the first year of sick leave and 70% in the second year. In general, episodes of sickness absence lasting for one or two weeks are self-certified. Employees in the Post and Telecommunication companies had to visit the occupational physician (OP) in the third week of sickness absence for medical certification. OPs certify mental disorders according to the 10^th ^International Classification of Diseases (ICD-10) [35]. The employer is responsible for the return to work process and the OP gives advice about the possibilities of return to work and work adjustment. The OP updates medical, social and vocational data in follow-up consults every four to six weeks, and guides sick-listed employees to return to work. Before 2004, a disability pension was granted after one year of sickness absence, but since 2004 a disability pension is granted after two years of sickness absence. For uniformity we applied the more conservative one year limit throughout the entire study period from 2001 to 2007, and censored the data of employees who were absent for longer than 365 days.

Data on episodes of medically certified sickness absence between January 1^st^, 2001 and December 31^st^, 2007 were retrieved from the records of ArboNed Occupational Health Services. We wanted to exclude employees who were absent at the start of the study, because these employees cannot be considered incident cases. Therefore, employees who were absent for more than 13 weeks due to sickness on 1 January 2001 were excluded, irrespective of the cause of the sickness absence. We choose this period, because employees who were absent for more than 13 weeks have to be reported to the social security agency in the Netherlands at that time and they were monitored by this agency during the course of their illness. We excluded 4158 employees due to this criterion. About 19% of them had common mental disorders. Sickness absence due to CMDs was medically certified as follows: distress (ICD-10 code R45), adjustment disorders (ICD-10 code F43), mild to moderate depressive disorders (ICD-10 codes F32.0 and F32.1), and anxiety disorders (ICD-10 codes F40.0, F40.1, F40.2, F41.0, F41.1, F41.2, and F41.3). We stratified the results into stress-related disorders (distress and adjustment disorders) and psychiatric disorders (depressive disorders and anxiety disorders).

### Statistical analysis

The first sickness absence episode due to CMD since January 1^st^, 2001 or the date of entering employment was regarded as the index episode in this study. The incidence density (ID) of index episodes was calculated by dividing the number of employees with a first episode of sickness absence due to CMDs between 2001 and 2007 by the person-years of the total population at risk. The recurrence density (RD) of sickness absence due to CMDs was computed by dividing the number of employees with recurrent episodes by the person-years in the sub-population of men and women with a previous episode of sickness absence due to CMDs in the different age groups. A recurrence was defined as the start of a new episode of absence due to CMDs after a recovery period of at least 28 days. We choose for this criterion, because in the Netherlands absences succeeding each other with an interval of less than 28 days are summed, in order to determine the qualifying period until work disability. The person-years at risk for ID were based on the total time of employment in the observation period. The person-years at risk for RD were based on the total time of employment in the observation period minus the time before the index episode of sickness absence due to CMDs.

**Figure 1:**
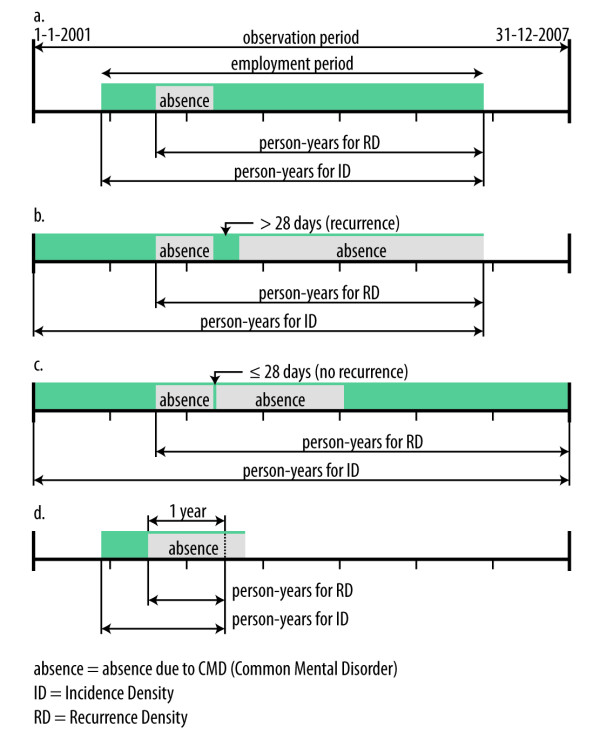
**Calculation of incidence density and recurrence density of sickness absence due to common mental disorders**.

Figure 1 shows the periods at risk for incidence and recurrence in different situations. In situation a) there is one episode of sickness absence due to CMDs and no recurrent episode. In that case, the total period of employment between January 1^st^, 2001 and December 31^st^, 2007 contributed to the person-years for the calculation of the ID. The person-years for the RD are counted from the beginning of the index episode of sickness absence due to CMDs until the end of the employment period. In situation b) a second absence due to CMDs occurs > 28 days after return to work. We define this situation as recurrent sickness absence due to CMDs. In situation c) there is a second episode of sickness absence due to CMDs within 28 days after return to work, which is not counted as a recurrence, because the Dutch social security legislation considers this situation as a connected episode of sickness absence. In the example, the employee is employed during the entire period. The person-years for the ID amount to seven years, and the person-years for the RD start at the beginning of the index episode of sickness absence due to CMDs. In situation d) there is an episode of sickness absence due to CMDs lasting more than 365 days. The person-years for the ID and the RD are counted until one year of sickness absence.

The ID and the RD of sickness absence due to CMDs were assessed in men and women in the age-groups of < 35 years, 35-44 years, 45-54 years, and ≥ 55 years. The median time-to-onset of a recurrent episode of sickness absence due to CMDs was computed with Kaplan-Meier survival curves. The survival curves for the age-groups were compared using the logrank test, based on the null hypothesis that the survival curves in the age-groups are the same [36].

## Results

### Descriptives of the population

Between 2001 and 2007, 8,951 employees (58% men and 42% women) had a total of 10,921 episodes of sickness absence episodes due to CMDs: 4,248 (39%) episodes with distress, 5,219 (48%) with adjustment disorders, 1,025 (9%) with depressive disorders and 429 (4%) with anxiety. In the period from 2001 to 2007, 7,379 employees (82%) had one episode of sickness absence due to CMDs; 1,274 (14%) had two episodes, 228 (3%) had three episodes and 70 (1%) had more than three episodes. The ID of sickness absence due to CMDs in the total population was 25.0 per 1000 person-years (95% confidence interval [CI] 24.5-25.5) between 2001 and 2007. After an episode of absence from work due to CMDs, the RD of sickness absence due to CMDs was 76.7 per 1000 person-years (95% CI 72.9-80.5). Of those with a recurrence, 90% experienced the recurrence within 3 years.

In the total study population the ID of stress-related disorders was 22.4 (95% CI 21.9-22.9) per 1000 person-years, which was higher than the ID for psychiatric disorders (3.7; 95% CI 3.5-3.9) per 1000 person-years. Table 1 shows the IDs stratified by age for men and Table 2 for women. After the index episode, stress-related disorders had an RD of 76.0 (95% CI 71.9-80.0) per 1000 person-years as compared to an RD of 81.6 (95% CI 70.8-92.5) per 100 person-years for psychiatric disorders. The RDs stratified for age are shown in Tables 1 and 2. In psychiatric disorders, the duration of a recurrent episode (72 days;95% CI 53-91 days) is shorter than the duration of the index episode (168 days; 95% CI 156-180) with p < 0.001, whereas in stress-related disorders the duration of a recurrent episode (51 days; 95% CI 46-56) did not differ from the duration of the index episode (53 days; 95% CI 51-55) with p = 0.221. The median duration until recurrence is 11 months for stress-related disorders (95% CI = 10-12 months) and 11 months (95% CI = 9-13 months) for psychiatric disorders (logrank test p = 0.913).

**Table 1:** Incidence and recurrence density of sickness absence due to common mental disorders by type of mental disorder in men of different ages

	Index episode			Recurrent episodes			Median time-to-onset recurrence in months (95% CI) ||
	Median duration			Median duration			
	N	in days (95%CI)	ID (95%CI)†	N	in days (95%CI)	ID (95%CI)†	
< 35 years	783	37 (33-41)	10.0 (9.3-10.8)	93	36 (18-54)	75.7 (61.8-89.6)	8 (3-13)
35 - 44 years	1554	53 (49-57)	22.2 (21.0-23.3)	270	47 (39-55)	75.0 (70.3-79.7)	12 (10-15)
45 - 54 years	1903	54 (50-58)	24.0 (23.0-25.1)	362	49 (40-58)	74.3 (70.8-77.8)	12 (10-14)
≥ 55 years	464	52 (46-58)	23.4 (21.6-25.2)	86	42 (27-57)	72.1 (58.1-86.0)	10 (8-12)

**Total men stress**	4704	49 (47-51)	19.7 (19.1-20.2)	811	46 (41-51)	74.4 (72.9-76.0)	11 (10-13)

< 35 years	127	153 (94-212)	1.6 (1.3-1.9)	14	25 (0-110)	70.0 (0-152.0)	14 (8-20)
35 - 44 years	257	168 (141-195)	3.6 (3.1-4.1)	48	81 (36-126)	97.0 (58.0-136.0)	8 (0-15)
45 - 54 years	286	178 (144-212)	3.7 (3.3-4.1)	55	103 (33-173)	80.1 (54.5-105.6)	14 (9-19)
≥ 55 years	53	171 (100-242)	3.0 (2.4-3.6)	9	31 (2-60)	73.9 (0-212.1)	8 (1-15)

**Total men psychiatric**	723	168 (150-186)	3.0 (2.8-3.2)	126	68 (39-97)	83.8 (71.9-95.7)	12 (8-15)

**Total men CMD**	34,603	57 (54-60)	21.8 (21.2-22.4)	937	48 (43-53)	75.6 (70.7-80.4)	11 (10-13)

† per 1000 person-years

|| in those with a recurrence

ID = incidence density

RD = recurrence density

CI = confidence interval

The table shows the age-distribution and the median duration (in days), ID and RD with their 95% CIs of sickness absence due to CMDs as well as the time-to-onset of recurrent sickness absence due to CMDs in months.

**Table 2:** Incidence and recurrence density of sickness absence due to common mental disorders by type of mental disorder in women of different ages

	Index episode			Recurrent episodes			Median time-to-onset recurrence in months (95% CI) ||
	Median duration			Median duration			
	N	in days (95%CI)	ID (95%CI)†	N	in days (95%CI)	ID (95%CI)†	
< 35 years	1016	49 (43-55)	24.3 (22.7-25.9)	151	62 (49-75)	94.4 (82.5-106.3)	9 (6-11)
35 - 44 years	1319	56 (51-61)	32.2 (30.5-34.0)	236	62 (47-77)	79.6 (73.7-85.5)	11 (9-13)
45 - 54 years	783	61 (54-68)	26.3 (24.6-28.1)	135	56 (42-70)	69.0 (60.6-77.3)	10 (7-13)
≥ 55 years	180	65 (48-82)	26.4 (23.0-29.9)	20	42 (0-129)	51.2 (15.3-87.2)	14 (13-15)

**Total women stress**	3298	56 (53-59)	27.8 (26.8-28.7)	542	60 (51-69)	78.4 (75.9-80.9)	11 (9-12)

< 35 years	212	153 (125-181)	5.1 (4.4-5.9)	28	73 (17-129)	90.4 (30.2-150.6)	9 (7-12)
35 - 44 years	242	161 (138-184)	5.8 (5.0-6.5)	44	85 (49-121)	90.3 (52.1-128.5)	10 (8-11)
45 - 54 years	136	217 (173-261)	4.9 (4.1-5.7)	18	67 (17-117)	53.5 (10.9-96.1)	11 (0-22)
≥ 55 years	22	248 (118-378)	3.2 (2.0-4.4)	3	59 (9-109)	66.1 (0-417.2)	12 (0-29)

**Total women psychiatric**	612	168 (151-185)	5.2 (4.7-5.6)	93	73 (53-93)	78.9 (64.1-93.7)	10 (8-12)

**Total women CMD**	18,026	67 (63-71)	31.5 (30.5-32.5)	635	62 (55-69)	78.5 (72.4-84.6)	10 (9-12)

† per 1000 person-years

|| in those with a recurrence

ID = incidence density

RD = recurrence density

CI = confidence interval

The table shows the age-distribution and the median duration (in days), ID and RD with their 95% CIs of sickness absence due to CMDs as well as the time-to-onset of recurrent sickness absence due to CMDs in months.

### Gender-specific ID, RD and time-to-onset of recurrence

As shown in Table 1, the median duration of sickness absence due to CMDs was 57 days (95% CI 54-60) in men, and sickness absence due to stress related disorders was of shorter duration than sickness absence due to psychiatric disorders. The ID of sickness absence due to stress-related disorders was 19.7 (95% CI 19.1-20.2) per 1000 person-years and the RD was 74.4 (95% CI 72.9-76.0) per 1000 person-years in men. The ID of sickness absence due to psychiatric disorders was 3.0 (95% CI 2.8-3.2) per 1000 person-years and the RD was 83.8 (95% CI 71.9-95.7) in men. The RD due to any type of CMDs after stress related and after psychiatric disorders did not differ significantly, because the 95% confidence intervals overlap. Recurrences were of shorter median duration than the index episode (48 days; 95% CI 43-53 days) but the survival curve for recurrent sickness absence did not differ significantly from the curve for the index episode (logrank test p = 0.172). The ID of stress-related and psychiatric disorders was lower in men < 35 years as compared to older men. The RD of sickness absence due to CMDs was not associated with age (logrank test p = 0.636), and the difference in time-to-onset of recurrence in men with a recurrence was not significant across age groups (logrank test p = 0.117). The median time-to-onset of recurrence in men was 11 months (95 CI = 10-13 months).

As shown in Table 2, the (median duration of sickness absence due to CMDs was 67 days (95% CI 63-71) in women and the shortest duration was found in the age-group of < 35 years (logrank test p < 0.001). As in men, sickness absence due to stress-related disorders was of shorter duration than sickness absence due to psychiatric disorders. The ID of sickness absence due to stress-related disorders was 27.8 (95% CI 26.8-28.7) per 1000 person-years and the RD was 78.4 (95% CI 75.9-80.9) per 1000 person-years. The ID of sickness absence due to psychiatric disorders was 5.2 (95% CI 4.7-5.6) per 1000 person-years and the RD was 78.9 (95% CI 64.1-93.7) per 1000 person-years. The RD due to any type of CMDs after stress related and after psychiatric disorders did not differ significantly, because the 95% confidence intervals overlap. The RD of sickness absence due to CMDs in women decreased with age (logrank test p = 0.004). Recurrences were of shorter median duration compared to index episode (62 days; 95% CI 55-69 days) but, as in men, the survival curve for recurrences did not differ from the curve for the index episode (logrank test p = 0.102). Recurrent sickness absence after stress-related or psychiatric disorders was most common in women < 35 years and between 35 and 44 years. The median time-to-onset of recurrence in women was 10 months (95% CI = 9-12 months). The difference in time-to-onset of recurrence in women with a recurrence was not significant across age-groups (logrank test p = 0.667).

Men had a lower ID of sickness absence due to CMDs than women (21.8 per 1000 person-years versus 31.5 per 1000 person-years), but the RD of sickness absence due to CMDs in men (75.6 per 1000 person-years) was almost the same as in women (78.5 per 1000 person years) with a logrank test result of p = 0.360.

## Discussion

The findings of this study show that after an episode of absence from work due to CMDs, the risk of recurrent sickness absence due to CMDs increases. Stress-related disorders had a higher incidence than psychiatric disorders, but the recurrence of CMD after stress-related disorders was not different from the recurrence of CMD after psychiatric disorders. Although sickness absence due to CMDs was more common in women, there were no gender differences in the recurrence of sickness absence due to CMDs. Recurrences were more frequent in women < 35 years and in women between 35 and 44 years. In men, we observed no difference in recurrence by age. Recurrent episodes of sickness absence due to CMDs were of shorter duration in men, but the median time-to-onset of a recurrence was comparable among women and men, and did not differ across age-groups.

In all, the results of this study confirm our hypothesis that the risk of recurrent sickness absence due to CMDs increases after a previous episode of sickness absence due to CMDs. The results indicate that recurrent episodes after sickness absence due to psychiatric CMDs were shorter than the index episode. Perhaps employees who have had a previous sickness absence episode due to psychiatric CMDs recognize their complaints and seek help at an earlier stage or rely on their previous experiences of returning to work after mental health complaints. It should be noted that in the present study the first episode of sickness absence due to CMDs since January 1^st^, 2001 was regarded as the index episode, regardless of any sickness absence before that date.

Women have a higher risk of CMDs than men [2,32-34], but gender was not related to the recurrence of major depressive disorder [6,37]. This contrasts the findings of Mueller et al., who reported that women had a higher recurrence of a major depressive disorder than men [5]. In our study population, women had longer episodes of sickness absence due to CMD than men and were censored more often at 365 days of sickness absence. When more men than women return to the population at risk, the incidence of recurrent sickness absence will be relatively higher among men. A recent meta-analysis showed conflicting results with regard to gender differences in return to work in employees with episodes of poor mental health [26]. According to De Rijk et al. [31], women did report a longer time until lasting return to work than men after a long-term absence due to mental disorders.

Younger age groups a have a higher risk of sickness absence due to CMDs [2,32-34]. We hypothesized that employees younger than 45 years have a higher risk of recurrent sickness absence due to CMDs than employees > 45 years. Our results demonstrate that the risks of recurrence in men do not differ significantly across the age groups. However, women under the age of 35 and between 35-44 years had higher recurrence of sickness absence due to CMDs than women between 45-54 years and ≥ 55 years of age. Nieuwenhuijsen et al. reported a poorer recovery from mental disorders in employees over 50 years of age [38]. Women ≥ 45 years with a poorer recovery might have been absent for a longer period of time and therefore had either a shorter risk period for a recurrence or a higher risk of being censored after 365 days of absence from work due to CMDs. The risk of recurrence in the oldest age groups may also have been underestimated, because older employees are more likely to resign or to be discharged, once long-term absent [39].

We found no significant difference across age-groups in the time-to-onset of recurrent sickness absence due to CMDs. Of the employees who had a recurrence, 90% experienced the recurrence within 3 years.

### Strengths and limitations

A strength of the present study is the use of sick-leave certificates issued by the OPs instead of self-reported complaints by the employees. However, the validity of psychiatric diagnoses on sickness absence certificates is a subject of ongoing scientific debate [17]. In a pilot study of 8,500 post sorters working for the post companies, all 546 employees who reported sick in 2003 consulted an OP and a psychiatrist [40]. Between OPs and psychiatrists, an agreement of 81% was found with regard to the group diagnosis of CMDs, confirming the results reported by O'Niell et al.[41]. However, the OPs frequently diagnosed distress or adjustment disorders, whereas the psychiatrists more often diagnosed depressive disorders and anxiety disorders.

The incidence of sickness absence due to CMDs was lower in our study population (25.0 per 1000 person-years) than the incidence of depressive and anxiety disorders in the general population in the Netherlands, which was 56.8 per 1000 person-years in 1999 [2]. The study population may be healthier than the general population and therefore have a lower incidence of depressive and anxiety disorders. It is also possible that employees with mild to moderate mental problems do not report sick. Depression and anxiety are known to be associated with presenteeism, i.e. being at work, but not able to perform adequately [4,5,42]. Alternatively, employees with mental disorders often present somatoform complaints [43,44], and in primary care it is not usual to ask detailed questions to discover underlying mental disorders [45].

Another limitation concerns the lack of data on co-morbidity. The sick-leave certificate only contained one diagnosis. Depression with comorbid disorders has been reported to have a higher likelihood of recurrence [15]. We are also not aware as to whether the diagnoses changed over time, which is a common shortcoming in studies of this type [17]. It may be that the RD person-years are over-estimated, because we used the time from the start of the index episode due to CMDs instead of the recovery date, whereas an employee who is on sick-leave is actually not at risk for recurrent sickness absence. However, the end date of an episode of sickness absence is less reliable than the start date, because episodes of sickness absence can end due to several reasons: return to work, leaving employment, the end of the company's contract with the occupational health service, administrative closure of the case after one year of sickness absence, or a change in the labour contract. Over-estimation of the person-years at risk in the denominator may have resulted in an under-estimation of risk of recurrence. The risk of recurrence may also have been under-estimated because of the high turnover in the study population. Employees who have been absent due to sickness are more likely to resign or to be discharged than those who have never been absent due to sickness [39]. The generous sick leave entitlements in the Netherlands may have influenced the tendency to report sick due to mental disorders and also the risk of recurrent sickness absence. Therefore, the results may not be generalized to other countries just like that.

The recurrence risk of sickness absence due to CMDs might in part be due to confounding by indication. If an employee is absent from work due to CMDs, the likelihood to be diagnosed CMD once absent from work a second time is much higher than to be diagnosed CMD a first time. This may have resulted in an over-estimation of RD due to CMDs.

The results of our studies of the Post and Telecommunications companies cannot be generalized to the entire working population in the Netherlands, because it has been reported that there are differences between companies in sickness absence policies and cultures [46]. However, in our population there are a considerable number of employees who carry out various types of work (heavy physical labour, back-office, technique, sales, IT, and executive functions) in a nationwide spread of establishments.

In conclusion, given the high recurrence rate of sickness absence due to CMDs and the median time of 10-11 months until recurrence, employees should be monitored after they have returned to work following sickness absence due to CMDs to prevent relapse. In particular, women under 35 years and between 35 and 44 years who are at high risk of recurrence should be monitored closely and offered regular preventive consultations. Future research should investigate the effects of interventions to prevent relapse of sickness absence due to CMDs or to facilitate sustainable return to work.

## Conclusions

Employees who have been absent from work due to CMDs are at increased risk of recurrent sickness absence due to CMDs and should be monitored after they return to work. Women under 35 years of age and between 35 and 44 years of age have a high risk of recurrence. However, because the risk of recurrence in women in other age-groups and men is also high, we recommend relapse prevention consultations for men and women in all age-groups.

## Competing interests

The authors declare that they have no competing interests.

## Authors' contributions

PCK, CAMR, JJL van der K and JWG were responsible for the conception and design of the study. PCK did the analysis and interpreted the analysis in collaboration with CAMR, UB and RH. PCK wrote the first draft of the manuscript. All authors critically revised the report for intellectual content and approved the final version of the manuscript.

## Pre-publication history

The pre-publication history for this paper can be accessed here:

http://www.biomedcentral.com/1471-2458/10/426/prepub
